# A retrospective study of CKDu progression in Sri Lanka: analysis of kidney biopsies and association with risk factors

**DOI:** 10.1186/s12882-026-05105-7

**Published:** 2026-06-06

**Authors:** Santhushya Hewapathiranage, Nilantha Pushpakumara, Thamalu Sonnadara, Thilini Weerakoon, Naduni Erandika, Sachintha Adhikari, Miriam Angeloni, Fulvia Ferrazzi, Kerstin Amann, A. W. M. Wazil, Rohana Chandrajith, Neelakanthi Ratnatunga, Sulochana Wijethunge, Philipp Enghard, Christoph Daniel, Nishantha Nanayakkara

**Affiliations:** 1 Centre for Research, National Hospital Kandy, Kandy, Sri Lanka; 2 Faculty of Engineering, University of Peradeniya, Peradeniya, Sri Lanka; 3 Institute of Pathology, Universitätsklinikum Erlangen, Friedrich- Alexander-Universität Erlangen-Nuernberg (FAU) Erlangen-Nürnberg, 91054 Erlangen, Germany; 4 Department of Nephropathology, Institute of Pathology, Universitätsklinikum Erlangen, Friedrich-Alexander-Universität (FAU) Erlangen-Nürnberg, 91054 Erlangen, Germany; 5 Nephrology and Kidney Transplant Unit, National Hospital, Kandy, Sri Lanka; 6 Department of Geology, Faculty of Science, University of Peradeniya, Peradeniya, Sri Lanka; 7 Department of Pathology, Faculty of Medicine, University of Peradeniya, Peradeniya, Sri Lanka; 8 Department of Nephrology and Medical Intensive Care, Charité Universitätsmedizin Berlin, Berlin, Germany

**Keywords:** CKDu, Kidney biopsies, Histopathology, Outcome prediction

## Abstract

**Background:**

Chronic kidney disease of uncertain aetiology (CKDu) is a regionally prevalent tropical disease whose pathogenesis is still unclear. Although kidney biopsies can help to characterize the disease, it is unclear whether histological analysis can also be used to make a prognosis.

**Methods:**

This retrospective study investigated kidney biopsy findings and progression factors in a 230 CKDu patients in Sri Lanka, with detailed follow-up of 62 participants for 4.2 ± 2.1 years.

**Results:**

Participants were predominantly male farmers (93.3%), and 87.0% reported agrochemical exposure. Histology showed a wide range of tubulointerstitial injury. Most specimens exhibited tubular atrophy (90.9%), interstitial fibrosis (75.2%), and glomerulosclerosis (92.1%) to varying degrees. Unsupervised hierarchical clustering of eight activity and chronicity variables identified three groups: (1) mild chronic changes with minimal inflammation, (2) pronounced inflammation with chronic changes, and (3) chronic changes with low grade inflammation. Estimated glomerular filtration rate correlated negatively with seven histologic parameters. Baseline eGFR was significantly higher in cluster 1 than in clusters 2 and 3. In the follow up cohort, tubulitis and glomerulosclerosis were associated with faster eGFR decline and predicted progression. Progression was not explained by differences in reported risk exposures.

**Conclusion:**

This large-scale biopsy study revealed that patients with CKDu could be categorised into three subgroups based on their histological changes, with significantly different eGFR values observed between these subgroups. Tubulitis was associated with faster decline in kidney function in the follow-up subcohort. Further research is needed to determine whether the identified clusters represent different stages of the disease or different progression.

**Supplementary Information:**

The online version contains supplementary material available at 10.1186/s12882-026-05105-7.

## Background

Chronic Kidney Disease of uncertain etiology (CKDu) has puzzled the scientific community since its emergence as a form of chronic kidney insufficiency among populations in tropical and subtropical regions, occurring in the absence of known causes such as diabetes and hypertension. In Sri Lanka, epidemiological data shows that the disease is concentrated remarkably around dry zone terrain and share common geographical pattern [1]. CKDu usually manifests in the age group 30–60 years, mostly in men from rice farming communities. Males are at higher risk than females. The disease is typically asymptomatic at onset with gradual progression to end-stage kidney disease (ESKD) over time. Characteristically, the histological changes are compatible with chronic interstitial nephritis, accompanied by early glomerular sclerosis [2, 3]. Numerous hypotheses have been proposed as possible causes of the disease, including the use of fertilizers, pesticides, herbicides, heavy metal contamination, fluoride, water hardness, and infections [1]. There is also evidence of an association between behavioral factors, such as dehydration and heat stress, with CKDu [2].

Moreover, the progression of CKDu to more advanced stages of kidney failure, associated with increased morbidity and mortality, has serious consequences for affected communities. Multiple factors may contribute to the accelerated progression of the disease. Hypertension has been identified as a major risk factor for the faster progression of CKDu, while continuous exposure to environmental risk factors may also play an important role [4]. A subsequent retrospective study reported that the progression of the disease varies among different patient categories. Younger individuals who presented at earlier stages of the disease progressed faster than other patients [4]. Microenvironmental exposures, particularly those related to drinking water sources, have also been associated with disease progression. The same study highlighted the dynamic nature of the community, noting that many individuals had switched their water sources to RO (reverse osmosis) water from their original sources [4]. Interestingly, in this community-based study, the group with faster disease progression was clustered in a lowland area, suggesting a possible link between geo-environmental factors and CKDu progression [4].

Kidney biopsies play a critical role in the study of CKD as they provide valuable insight into the structural and pathological changes in kidney tissue and are essential for ruling out other kidney diseases [5]. Histopathologic changes in CKDu have been described from patients in different endemic regions, but most rely on a limited number of cases [6]. Only a few studies have examined cohorts of more than 200 patients and described CKDu [7, 8]. However, in CKDu kidney biopsy findings are non-specific and predictive value has not been scientifically evaluated [9–11]. Histological lesions in CKDu are characterized by irreversible changes, such as tubular atrophy, interstitial fibrosis, glomerular sclerosis, and periglomerular fibrosis, as well as reversible changes, including tubular interstitial cell infiltration and tubulitis. Histologic evidence of chronicity and activity in a kidney biopsy can be assessed using various parameters and is therefore prognostically important.

The present study was carried out to investigate the spectrum of kidney biopsy findings in CKDu patients and to evaluate whether unsupervised clustering analysis of 230 CKDu patients could identify, based on histological evidence of chronicity and activity, subgroups with different histologic patterns. In a sub cohort of patients with follow-up data we investigated possible associations between the histological pattern and patient behaviors, risk factors and disease progression.

## Materials and methods

### Data collection

This retrospective study included patients who underwent kidney biopsy between 2012 and 2019 and resided in CKDu-endemic regions of the North Central, Central, and Uva provinces of Sri Lanka. A total of 2,766 kidney biopsy samples, stored in the Department of Pathology at the University of Peradeniya, were screened for inclusion in the study. Of these, 230 cases were identified as having CKDu based on clinicopathological correlations. The criteria for classifying cases as CKDu included a histopathological diagnosis of chronic interstitial nephritis with exclusion of immune-mediated and secondary causes, together with clinical exclusion of other recognized causes of chronic kidney disease, including diabetes mellitus, significant hypertension, vascular disease, prior nephrotoxic drug exposure, previous kidney infection, obstructive uropathy, and nephrolithiasis as a primary cause of chronic interstitial nephritis.

Kidney biopsy had been considered only in patients younger than 70 years, with kidney length greater than 9 cm in each kidney, and serum creatinine above the upper limit of normal, defined as more than 1.2 mg/dL on two consecutive assessments, irrespective of urinary abnormalities. Biopsies with fewer than 7 glomeruli on light microscopy were excluded. Cases with kidney stone disease were also excluded from the CKDu analysis set. Final inclusion in the present study required clinicopathological features consistent with CKDu after exclusion of immune-mediated and secondary causes of chronic kidney disease. Patients whose findings were considered to represent isolated acute kidney injury were not included in the final cohort.

Before biopsy, all patients were assessed clinically by two independent nephrologists. Patients with clear evidence of a secondary cause for chronic interstitial nephritis were not classified as having CKDu.

### Histopathological assessment

**Fig. 1:**
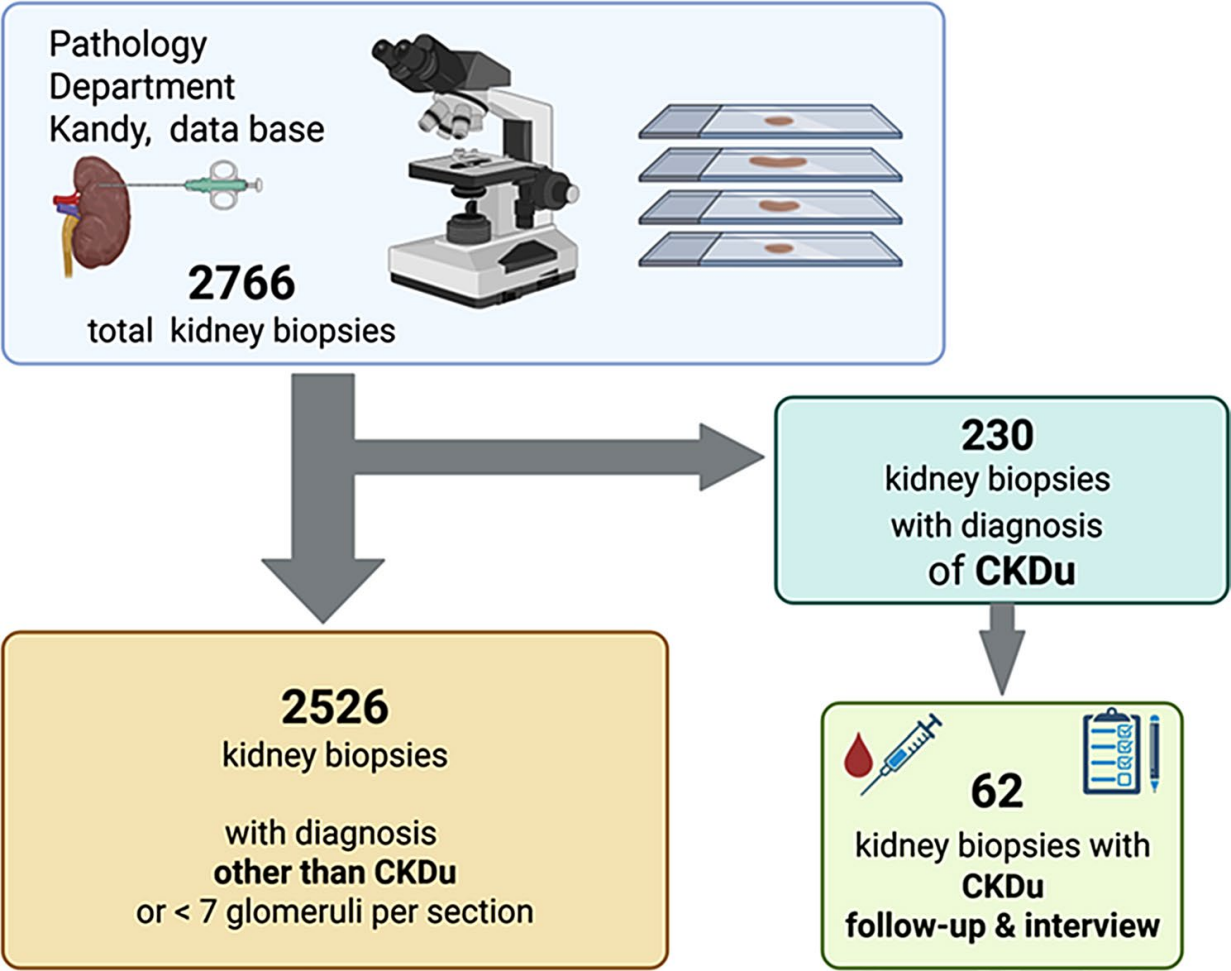
Selection of CKDu cases from the kidney biopsy database of the Department of Pathology, Peradeniya University. Created in BioRender.com

Histological findings compatible with primary tubulointerstitial nephritis include absence of significant glomerular pathology except global glomerulosclerosis, together with tubular atrophy, tubulitis, lymphocytic infiltration, periglomerular fibrosis, and interstitial fibrosis, with or without global glomerulosclerosis [8, 12] (Fig. 1). These features were present in variable degrees depending on the activity and chronicity of the disease. Cases with the diagnosis of primary glomerular diseases were excluded. Histological assessment was performed on formalin-fixed paraffin-embedded sections with hematoxylin and eosin (HE) staining, relevant special stains, and immunofluorescence studies with IgG, IgA, IgM, and C3 using the direct method. The minimum number of glomeruli required for histopathological assessment was seven, and for immunofluorescence, one glomerulus. Accordingly, all biopsies included in the study were considered adequate for light microscopic assessment, and immunofluorescence findings were reviewed when available.

The kidney histological parameters collected were glomerular sclerosis as a percentage of the total glomeruli present, tubulitis, tubular atrophy, interstitial inflammation mainly composed of lymphocytes and plasma cells, interstitial fibrosis, and periglomerular fibrosis. These parameters were scored according to visual analysis on a scale of 0 to 3, with 0 indicating no kidney lesions, 1 indicating up to 30% of the kidney surface involved, 2 indicating 30 to 60% involvement, and 3 indicating more than 60% involvement. The activity index was scored as the sum of interstitial lymphocyte infiltration and tubulitis (score 0 to 6), and the chronicity index as the sum of glomerular sclerosis, tubular atrophy, interstitial fibrosis, and periglomerular fibrosis (score 0 to 12). The activity score was categorized as mild, moderate, and severe as follows: 2 as mild, 4 as moderate, and 6 as severe. Similarly, the chronicity index was categorized as 1 to 4 as mild, 5 to 8 as moderate, and 9 to 12 as severe. This classification was based on the standardized scoring system used by the Department of Pathology, University of Peradeniya, for assessing cases with CKDu, as described earlier [12] Tubulitis was defined as the presence of inflammatory cells within or between tubular epithelial cells. Inflammatory cells located around tubules but not infiltrating the tubular epithelium were classified as interstitial inflammation. In the original scoring system, tubulitis and interstitial inflammation were recorded according to morphologic extent whenever present, irrespective of coexisting chronic changes such as tubular atrophy or interstitial fibrosis. However, inflammation confined to fibrotic areas and around atrophic tubules was not interpreted as unequivocal evidence of ongoing active injury. Because the original scoring system did not separately record tubulitis in preserved versus atrophic tubules, a compartment-specific analysis of tubulitis was not possible and is acknowledged as a limitation. Tubulitis and interstitial inflammation were included in the activity score as morphologic indicators of inflammatory involvement within the tubulointerstitial compartment. However, their presence was interpreted in the context of accompanying chronic structural changes. In contrast, inflammation present within fibrotic areas and around atrophic tubules was interpreted as a chronic sequela of a prior insult rather than as evidence of ongoing active injury. Therefore, inflammatory changes were interpreted in the context of the overall biopsy pattern rather than as isolated markers of activity.

Sixty-two out of 230 CKDu patients (Fig. 1) were contactable and interviewed with a detailed questionnaire to collect demographic data, farming history, agrochemical use and exposure levels, drinking water sources, tobacco and alcohol use, snake bites, family history of CKD and history of kidney stones. Renal function trajectory in the follow-up subgroup was assessed using serial eGFR measurements obtained from clinical records. The annual change in eGFR was calculated from the available longitudinal data and was used for correlation analyses with histopathological parameters. For descriptive comparison of patients with relatively faster and slower decline, a threshold of > 3 mL/min/1.73 m²/year was used to define the more rapidly declining subgroup.

Proteinuria was assessed at the time of biopsy using dip-stick analysis in the total study cohort (*n* = 230). The eGFR was calculated using the 2021 CKD-EPI formula.

The participants’ blood pressure (mm/Hg) and serum creatinine (mg/dL) levels from the day of the biopsy to the day of the interview were obtained from their medical records. Informed consent was obtained from each patient. Ethical approval for the study was obtained from the National Hospital, Kandy, Sri Lanka.

## Statistical analyses

Multivariate analysis of variance (MANOVA) was performed to identify associations between the histological scores and eGFR decline. Associations between demographic and behavioural characteristics and disease progression, defined as an eGFR decline of more than 3 mL/min/year, were investigated among the interviewed participants. Correlations between histological variables and follow-up kidney function were assessed using Spearman’s correlation test, with annual eGFR change as the follow-up outcome variable. For all the above-mentioned statistical analyses, SPSS statistics software version 28 (IBM, Armonk, NY, USA) was used.

Unsupervised hierarchical clustering was performed in R v4.2.2 [14]. The input data consisted of.

8 variables for 230 CKDu patients, which measured glomerular sclerosis, interstitial fibrosis, tubular atrophy, chronicity index, periglomerular fibrosis, tubulitis, lymphocyte infiltration, and activity index. Hierarchical clustering was performed through the R function hclust, using Manhattan distance for distance matrix computation and ward. D2 as agglomeration method. Heatmap visualization of hierarchical clustering results relied on the function pheatmap v1.0.12.

A top colour ribbon was added to the heatmap as further annotation indicating the eGFR class associated with each patient. Differences in the distribution of eGFR across the identified CKDu clusters were assessed relying on the Kruskal-Wallis test using the function Kruskal test() from the rstatix R package v0.7.2. Pairwise differences between clusters were assessed through the Dunn’s test using the dunn_test() function from rstatix. P-values were adjusted for multiple testing using the Bonferroni correction.

A p-value (p) or an adjusted p-value < 0.05 was considered statistically significant.

## Results

### Characterization of the CKDu cohort

**Fig. 2:**
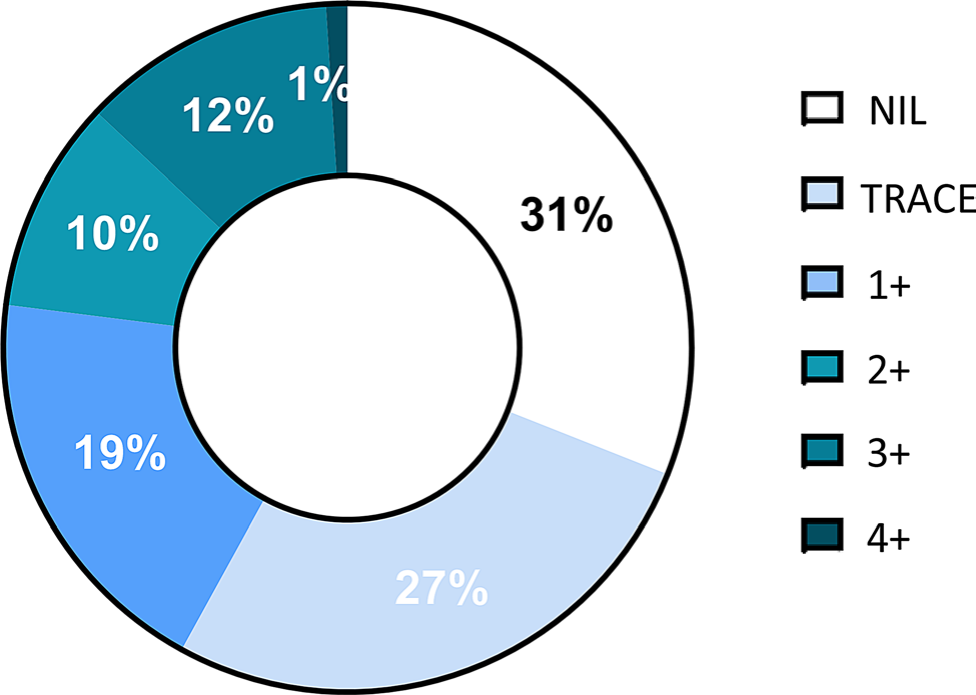
Distribution of proteinuria in the CKDu study cohort in percent. Proteinuria was determined at the time of biopsy using dip-stick analysis in the total study cohort(*n* = 222)

The histological features observed in this biopsy cohort were broadly consistent with those previously described in CKDu [8]. The study population showed tubular atrophy, interstitial fibrosis, periglomerular fibrosis, glomerular sclerosis, interstitial inflammation and tubulitis of variable degrees (Table 1). Most cases showed variable degrees of chronic changes, such as tubular atrophy and interstitial fibrosis, with or without global glomerular sclerosis, often accompanied by ongoing inflammatory activity. In the majority of cases, active disease characterised by lymphocyte-mediated tubulitis was mild (57.8%) or moderate (21.7%) and absent (16.5%) in a significant proportion (Table 1). Hypertensive vasculopathy was observed in the context of advanced chronic changes, suggesting that it represents a secondary manifestation rather than primary hypertensive kidney disease. The only glomerular change observed was global glomerular sclerosis. On light microscopy, the viable glomeruli did not show glomerular enlargement or ischemic-type capillary wall wrinkling, and the remaining glomerular tufts were otherwise unremarkable. Periglomerular fibrosis was frequently observed in conjunction with interstitial fibrosis. Glomeruli were negative for immune deposits. Apart from age and slightly higher eGFR, all the other characteristics were comparable between the main group and the interviewed subgroup. The majority of the patients did not have significant proteinuria (NIL-Trace) consistent with a diagnosis of CKDu (Fig. 2). However, at least 13% of the study population had significant proteinuria (3 + or more), regardless of their biopsy evidence of primary interstitial nephritis (Fig. 2). In these cases, coexisting glomerular diseases, such as minimal change disease, focal segmental glomerulosclerosis, and other primary glomerular pathologies, were evaluated using clinical, histopathological, and immunofluorescence findings. None of the patients with 3 + or 4 + proteinuria had edema or hypoalbuminemia suggestive of nephrotic syndrome. In addition, hematuria suggestive of an underlying glomerular basement membrane abnormality was not identified in this subgroup. However, as electron microscopy was not performed, minimal change disease or another coexisting pathology cannot be excluded, and significant proteinuria in these patients should be interpreted with caution.

**Table 1:** Demographic data and disease progression patterns of the total study cohort and the interviewed cohort

	Total study cohort (*N* = 230) Mean ± SD (range) │ *N* (%)	Interviewed cohort (*N* = 62) Mean ± SD (range) │ *N* (%)
Age	45.8 ± 11.1 (15.0–70.0)	48.0 ± 8.9 (25.0–64.0)
Gender		
Male	195 (84.78)	58 (93.55)
Female	35 (15.22)	4 (6.45)
Residence	NA	
Central province	NA	17
North central province	NA	20
Uva province	NA	24
Serum Creatinine (mg/dL)	2.2 ± 2.5 (0.6–34.1)	1.8 ± 0.6 (1.0-4.5)
eGFR (mL/min/1.73m2)	47.0 ± 23.7 (1.0-125.2)	48.0 ± 15.6 (13.7–90.4)
eGFR decline (mL/min/year)	NA	0.0060 ± 0.75 (-3.83 to 2.55)
CKDu stage category		
Stage 1	16 (6.96)	1 (1.61)
Stage 2	32 (13.91)	11 (17.74)
Stage 3	133 (57.83)	44 (70.97)
Stage 4	35 (15.22)	3 (4.84)
Stage 5	14 (6.09)	2 (3.23)
Histological disease Activity index		
Null	38 (16.52)	9 (14.52)
Mild	133 (57.83)	36 (58.06)
Moderate	50 (21.74)	15 (24.19)
Severe	9 (3.91)	2 (3.23)
Histological disease Chronicity index		
Null	2 (0.87)	1 (1.61)
Mild	115 (50.00)	31 (50.00)
Moderate	107 (46.52)	27 (43.55)
Severe	6 (2.61)	3 (4.84)

### Unsupervised hierarchical clustering identifies 3 CKDu patients’ subgroups with distinct histopathological changes

**Fig. 3:**
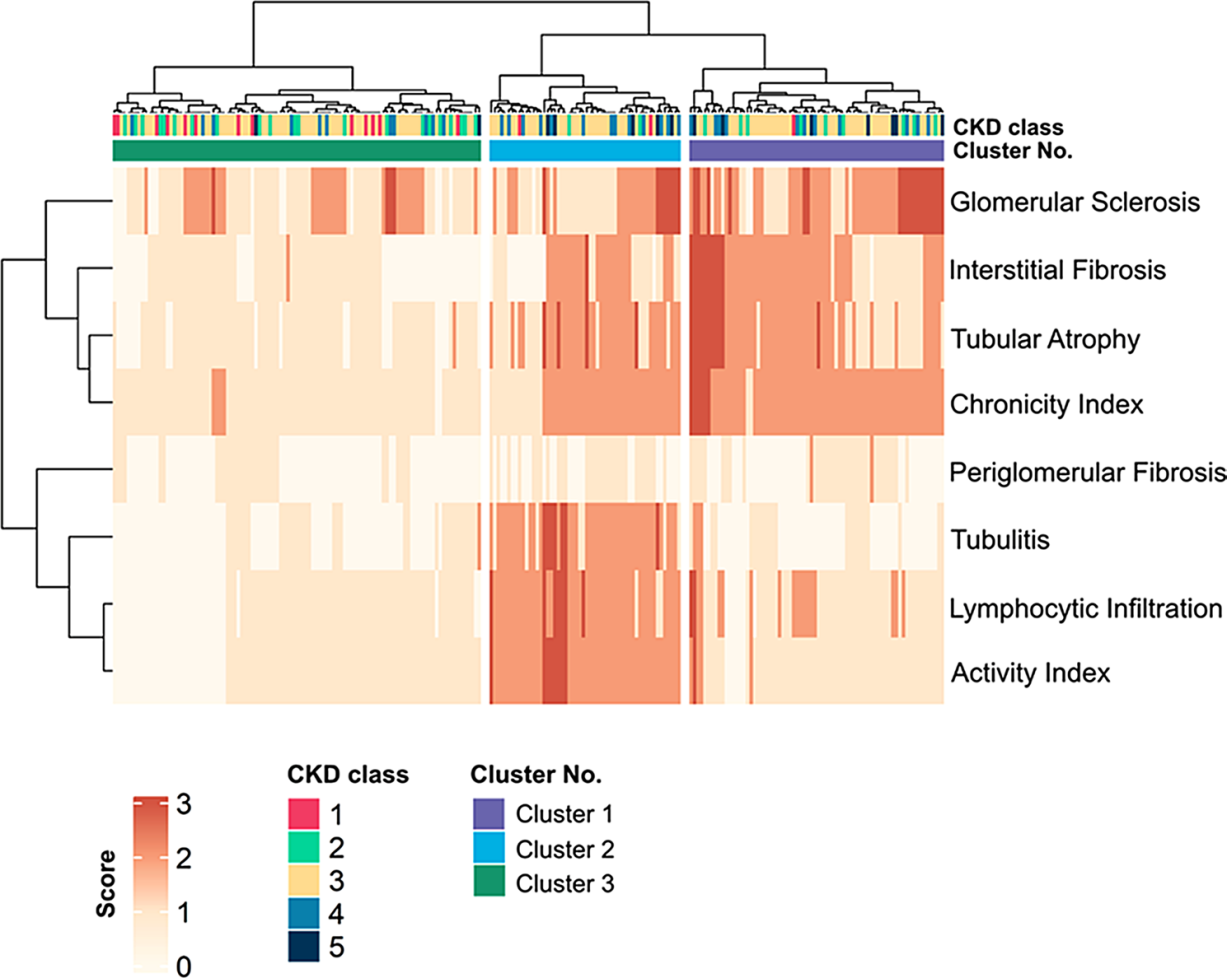
Unsupervised hierarchical clustering of 230 CKDu cases using histopathological changes. Heatmap visualization of the hierarchical clustering analysis performed on the 8 activity and chronicity markers in the 230 CKDu samples. Heatmap colors represent markers’ scores: the darker the color, the more severe the marker change. The color ribbons at the top of the heatmap indicate: the three identified subgroups of patients (bottom) and the eGFR class associated with each patient (top)

**Fig. 4:**
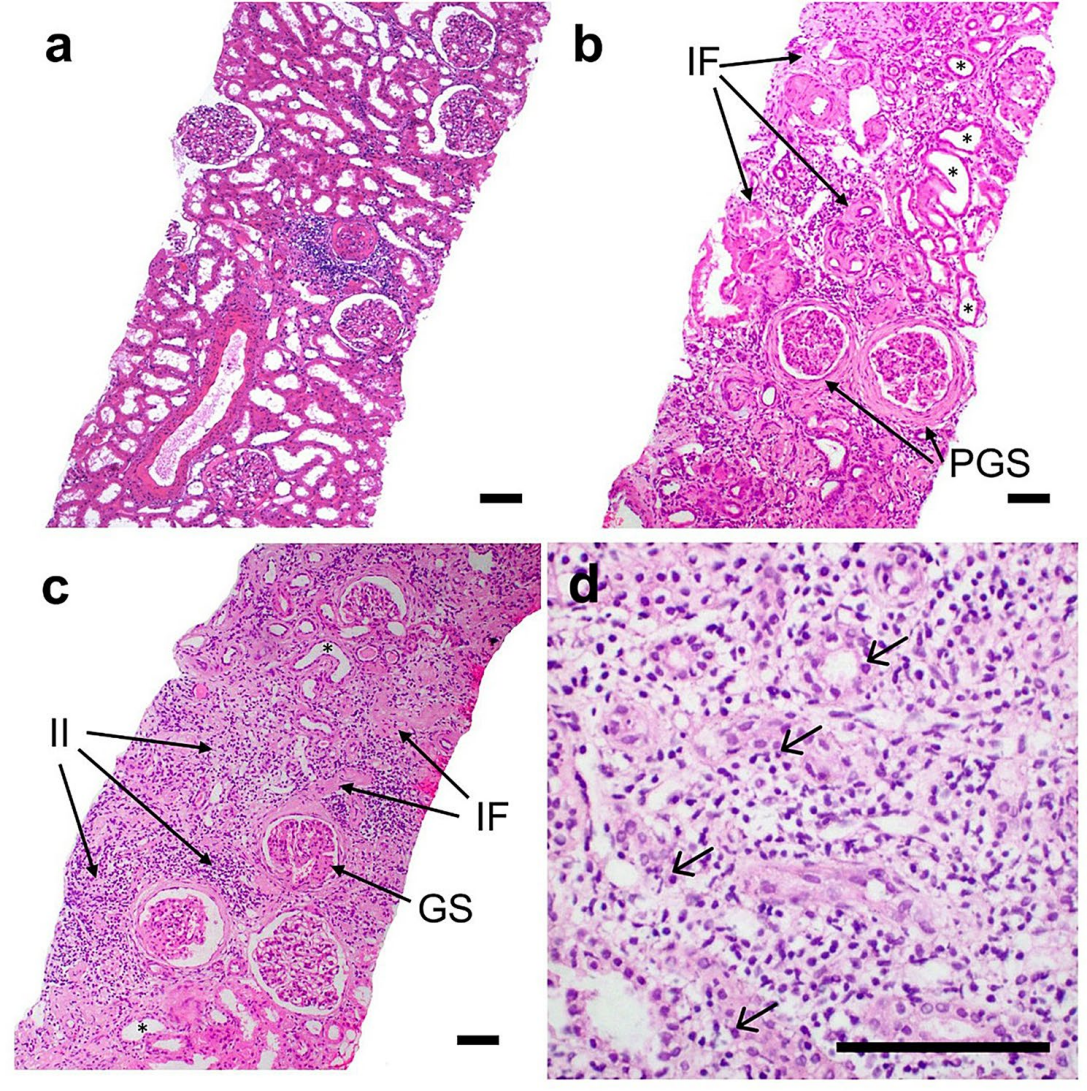
The spectrum of histopathologic changes in a Sri Lankan CKDu cohort with 230 patients. Representative images of hematoxylin/eosin-stained sections diagnosed with CKDu. **a**: Example of a case from cluster 1 with mild inflammation and lacking interstitial fibrosis **b**: Example of a case from cluster 2 characterized by mild to moderate inflammation and significant interstitial fibrosis (IF). Periglomerular fibrosis (PGF) is not restricted to cluster 2 with most advanced chronic lesions. **c**: Example case from cluster 3 showing both significant interstitial inflammation (II) and interstitial fibrosis (IF). Glomerulosclerosis (GS) is more prevalent in the example biopsies from clusters 2 and 3. **d**: Tubulitis, marked by black arrows, is an activity marker and a common finding in most CKDu biopsies. Scale bars represent 100 μm

When considering the splitting with three clusters, unsupervised hierarchical cluster analysis of the 230 CKDu patients based on chronicity- and activity-related scores revealed patients´ subgroups with distinct patterns (Fig. 3). In the first cluster, the CKDu biopsies show mild to moderate chronic changes such as tubular atrophy and interstitial fibrosis with absent to mild inflammation (Fig. 3, purple labeling). In this cluster, the variable glomerulosclerosis varies across patients. The second cluster is characterized by chronic changes with marked interstitial fibrosis and tubular atrophy, but only mild inflammation (Fig. 3, purple labeling). In the third cluster, the biopsies show pronounced chronic changes as well as a marked inflammation with tubulitis and lymphocyte infiltration (Fig. 3, light blue labeling). The variable periglomerular fibrosis appears uniformly distributed across the 3 clusters, but least pronounced in cluster 1 (Fig. 3). Pairwise correlation analysis within the activity scores (i.e., activity index, tubulitis, and lymphocytic infiltration) resulted in significant results, with positive correlation coefficients higher or equal than 0.606 (Supplementary Fig. 1). Significant correlation results also emerged when performing pairwise correlation analysis within the chronicity scores (i.e., chronicity index, tubular atrophy, and interstitial fibrosis), with positive correlation coefficients higher than 0.679. Pairwise correlation analysis between activity markers and chronicity markers also led to significant, positive correlation results, as for example between lymphocytic infiltration and tubular atrophy with a correlation coefficient of 0.496 (Supplementary Fig. 1). Instead, negative correlation results were observed between histologic parameters and eGFR (Supplementary Fig. 1). Examples of hematoxylin and eosin-stained biopsy specimens, each representative of one cluster, are shown in Fig. 4. A kidney biopsy belonging to cluster 1 shows only mild interstitial fibrosis and mild tubular atrophy (Fig. 4a). In contrast, a biopsy from cluster 2 shows pronounced interstitial fibrosis (IF) as well as tubular atrophy, mild inflammation, and periglomerular fibrosis (PGF) (Fig. 4b). Glomerular sclerosis is rather mild in this case (Fig. 4b). The example biopsy from cluster 3 shows both marked infiltration with inflammatory cells (Fig. 4c, II) and tubulitis (Fig. 4d), but also signs of chronic changes such as interstitial fibrosis, tubular atrophy and glomerular sclerosis (Fig. 4c).

### Biopsies characterization with eGFR

**Fig. 5:**
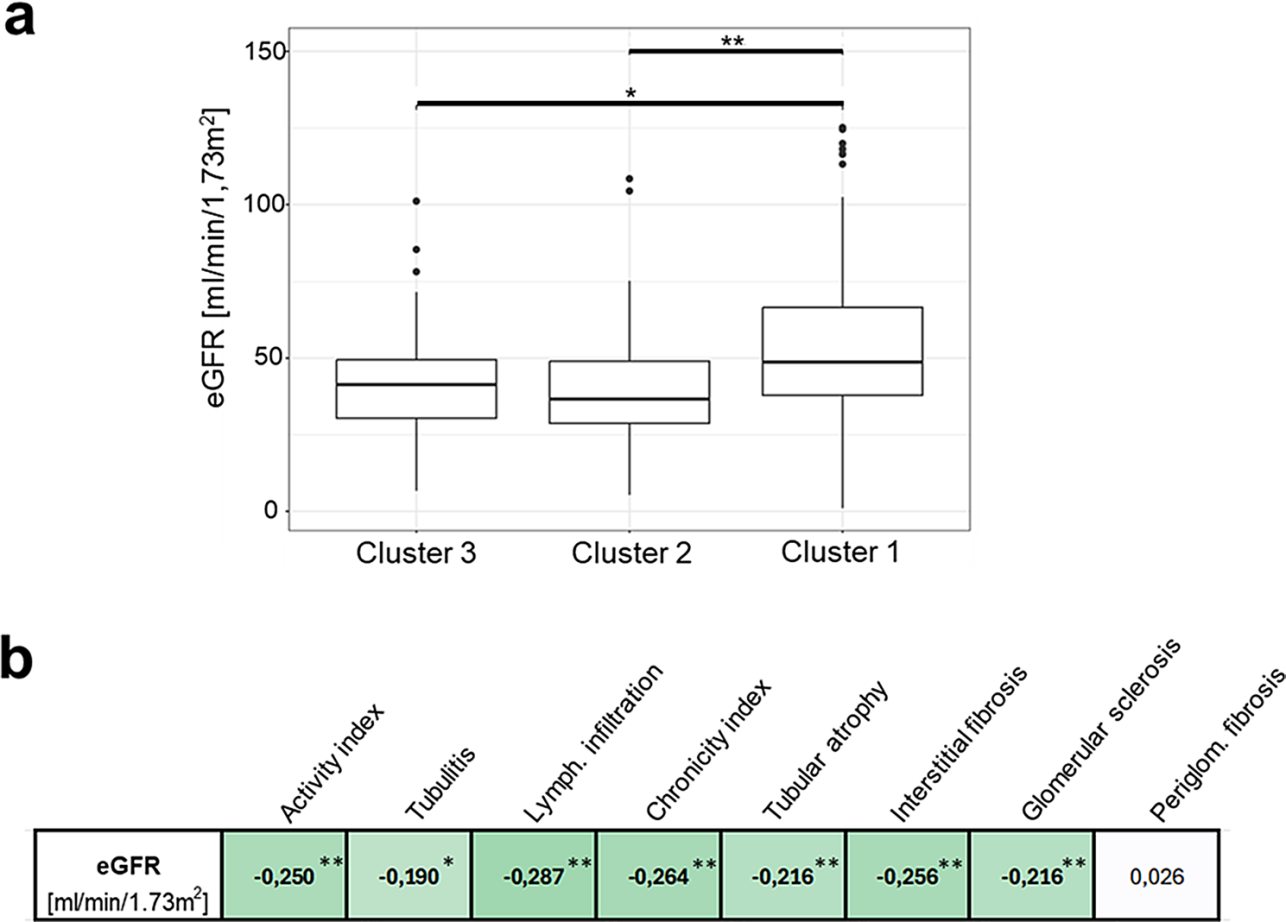
Distribution of eGFR in clusters 1–3 and correlation with histopathologic lesions. **a**: Boxplot distributions of eGFR in the identified clusters. P-values are from Dunn’s post-hoc test. **b**: Spearman’s correlation coefficients between eGFR and histopathological lesions. *p < 0.01; **p < 0.001

The identified clusters were characterized in terms of kidney function by analyzing their association with eGFR. A significant association was found between the 3 clusters and eGFR (*p* = 6e-05). Cluster 1, which had a lower chronicity index, showed the highest median eGFR, whereas Cluster 3, with a higher chronicity index, had the lowest median eGFR (Fig. 5a). When performing correlation analysis between eGFR and each chronicity-/activity-related variable used for hierarchical clustering, significant negative correlation results were found, except for periglomerular fibrosis (Fig. 5b). Correlation coefficients for significant association results ranged from − 0.190 to -0.287 (Fig. 5b), thus indicating that the correlation, although significant, is rather weak. Therefore, according to our results, serum creatinine alone may not reflect the temporal progression of CKDu but rather represents the underlying pathogenic processes, highlighting the relevance of kidney biopsy.

**Fig. 6:**
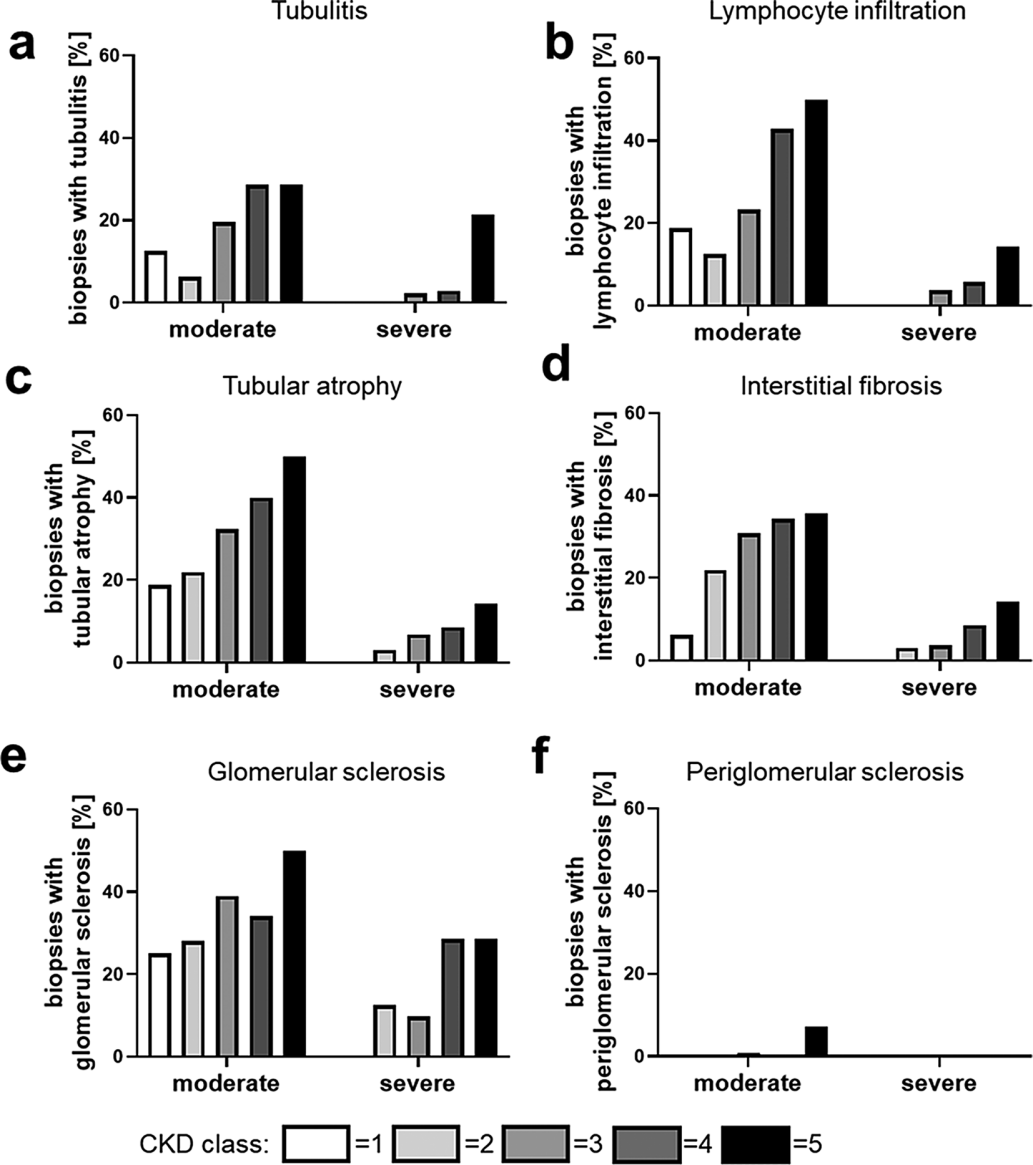
Frequency of moderate and severe histopathologic lesions in CKD classes. Distribution of CKD classes across the subset of patients with moderate (score 2) and severe (score 3) **a**: tubulitis, **b**: lymphocyte infiltration, **c**: tubular atrophy, **d**: interstitial fibrosis, **e**: glomerular sclerosis, **f**: periglomerular sclerosis

When examining the frequency of severity of histologic changes across CKD classes 1–5, we observed a spectrum of different degrees of lesion severity within each CKD class (Supplementary Fig. 1). Although both mild and severe degrees of histopathologic changes were observed in all CKD classes, moderate to severe lesions of the activity markers tubulitis (Fig. 6a) and lymphocyte infiltration (Fig. 6b) as well as chronicity markers such as tubular atrophy (Fig. 5c), interstitial fibrosis (Fig. 6d) and glomerulosclerosis (Fig. 6e) increased in patients with more severe CKD and lower eGFR. Only the frequency of periglomerular fibrosis was independent of CKD stage (Fig. 6f). According to the multivariate analysis (M-ANOVA) the scoring values of histopathological lesions were significant between CKD stages *P* = 0.013 (pillai’s trace), *P* = 0.017 (wilks lambda test), which confirms the correlation results between eGFR and histological parameters (Fig. 5b). In conclusion, kidney function as assessed by eGFR is affected by histopathologic lesions, but not to the extent that eGFR is sufficient to accurately predict the underlying pathologic process in CKDu.

### Tubulitis is associated with eGFR decline in the follow-up subcohort

**Fig. 7:**
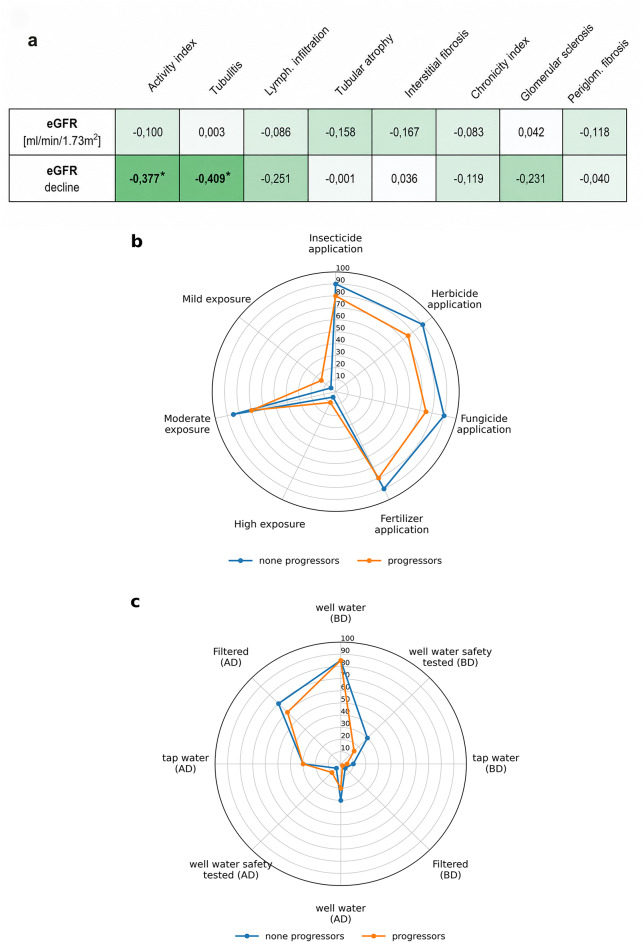
Correlation of eGFR decline with histopathologic lesions and patterns of agrochemical use and water supply in slowly and rapidly progressive disease. **a**: Spearman’s correlation coefficients between: (top) eGFR and histopathological lesions, and (bottom) eGFR decline and histopathological lesions in a follow up subcohort of 62 cases. * indicates *p* < 0.05. **b**: Various types of agrochemical usage pattern and exposure levels among the participants. (Exposure levels: High –Agrochemical applicator for their own land and others lands, Moderate- Agrochemical applicator only for their own land, Mild: non-applicator with secondary exposure). **c**: Comparative usage of drinking water resources by the participants before diagnosis (BD) and after the diagnosis (AD) of CKDu. Group sizes: none progressors *n* = 33, progressors *n* = 29

Out of 230 CKDu patients, only seventy-five were contactable for the retrospective data collection during the study period. The mean follow-up duration from biopsy was 4.2 ± 2.1 years, during which eGFR and serum creatinine data were available for analysis. To determine whether individual histologic changes could predict progression of CKDu, measured as eGFR decline, correlation analyses were performed in the follow-up cohort of 62 patients. In this smaller sub-cohort, the correlations between eGFR at the time of biopsy and the severity of the various kidney lesions were not significant (Fig. 7a). In the follow-up subcohort, the mean annual change in eGFR was − 0.0060 ± 0.75 (range, -3.83 to 2.55). Two patients showed an eGFR decline of more than 3 mL/min/1.73 m²/year. However, eGFR decline significantly correlated with tubulitis (Fig. 7a). This indicates that this histologic change associates with the disease progression. Despite a smaller sample size, pairwise correlations between histologic parameters remained highly significant at a level comparable to the overall cohort (Supplementary Fig. 2).

A notable percentage of 93.3 from the interviewed cohort were engaged in farming, 87.0% of which were using various kinds of agrochemicals. Since the histological characterization of the initial kidney biopsy did not provide information on the progression of kidney function loss, we investigated whether the source of drinking water or the patients’ exposure to agrochemicals influenced the progression.

Evaluation of the sources of drinking water used by patients with CKDu showed that they initially used mainly well water and switched to other safer sources such as RO water after diagnosis. However, progressors used comparable sources of drinking water, so we could not demonstrate that rapid progression of CKDu is due to a deterioration in drinking water quality (Fig. 7c).

## Discussion

In this study, we analyzed kidney biopsies from 230 patients with CKDu and characterized the spectrum of histopathological lesions. Using unsupervised hierarchical clustering, three distinct subgroups were identified based on activity and chronicity markers. The severity of histological changes showed a weak correlation with eGFR. Given the weak correlations between histological features and eGFR, kidney function alone may be insufficient to assess the extent of activity, chronicity, and temporal progression of kidney pathology in CKDu; therefore, kidney biopsy continues to play an important role in its comprehensive evaluation.

A spectrum of histological changes, predominantly reflecting tubulointerstitial injury, has been described in earlier biopsy studies from Sri Lanka [7, 8, 12, 15, 16] and other tropical countries where CKDu occurs [17–19] including a recent series from India that reports the same combination of glomerulosclerosis, glomerular hypertrophy, and mild-to-moderate interstitial fibrosis and tubular atrophy (IFTA) [20]. Our results from more than two hundred kidney biopsies clearly describe the characteristic light microscopic abnormalities in CKDu. According to our results, the majority of our study participants had both acute and chronic lesions in their biopsies. In this study, through the application of unsupervised hierarchical clustering, CKDu patients were divided into well-defined subgroups, each of which characterized by distinct histological features. Three clusters were found, showing either only mild activity and chronicity (cluster 1), predominantly chronic changes (cluster 2), or both marked activity and chronicity (cluster 3). However, it is still unclear whether the clusters we found reflect different stages during the progression of CKDu or else subtypes of the disease related to severity of exposure [21, 22].

Histopathological studies of CKDu in large patient cohorts remain limited, despite the important role of kidney biopsy in understanding disease pathogenesis. Previous smaller studies have described a spectrum of histological changes in CKDu biopsies. In endemic populations, tubulointerstitial disease in the absence of classical CKD risk factors is considered the diagnostic hallmark of CKDu. However, in our cohort, a small proportion of participants presented with significant proteinuria. As specific kidney lesions attributable to proposed nephrotoxins have not yet been clearly defined, identifying a single causative agent based solely on histological findings is not currently feasible. Accordingly, caution is warranted when attributing CKDu to specific exposures based on biopsy findings alone. Nevertheless, defining the characteristic histological features of CKDu remains highly relevant, as it provides a foundation for future identification of toxin-specific lesions [22, 23].

Glomerular sclerosis was observed in 92.1% of subjects with increasing severity in advanced disease, nevertheless, tubulitis (48.2%) and lymphocytic infiltration (81.3%) were among consistent findings. In a previous study of 43 diagnosed cases of CKDu [2] only 15% of the study cohort had global glomerular sclerosis. Contradictorily, another study of 125 patients reported 94.8% of glomerular sclerosis [16]. Several other studies have reported that the severity of global glomerulosclerosis tends to increase progressively with CKD stage [12, 16, 19]. A similar pattern of combined chronic glomerular and tubulointerstitial injury has also been described in Mesoamerican nephropathy, with 19 patients in which kidney biopsies showed glomerulosclerosis, glomerular hypertrophy, signs of chronic glomerular ischemia, and only mild to moderate tubulointerstitial damage [18]. This observation was confirmed in our study. Although global glomerulosclerosis was significantly negatively correlated with eGFR, and the proportion of biopsies with moderate and severe glomerulosclerosis increased with higher CKD classes, the correlation was at a low level, which again emphasizes the limited association of eGFR with histological changes.

Almost all biopsies showed at least mild signs of tubular atrophy as a sign of chronic changes, and one third of biopsies in cluster 1 showed no fibrosis at all. The coexistence of active inflammation and well-established chronic histological changes may indicate either early irreversible damage or recurrent insults in already damaged kidneys. Cluster 2 may represent an advanced stage in the pathogenesis of CKDu, as the inflammatory response is largely resolved by this time. The mild changes in cluster 1 suggest that these biopsies represent an early form of CKDu, whereas the other two clusters represent more advanced stages of CKDu. This hypothesis is supported by the significant association found between the identified clusters and eGFR, with eGFR values in cluster 1 higher than in the other two clusters. Another important finding of this study was that eGFR did not accurately reflect histological changes in the kidney, as it showed poor correlation with individual histopathological features. Notably, Cluster 1 also included patients with low eGFR. This discrepancy between kidney function and histological lesions may be explained by the combined effects of acute and chronic changes. In the presence of acute interstitial nephritis, kidney function could be severely impaired without remarkable light microscopic changes. Conversely, in chronic interstitial disease, glomerular hyperfiltration and other compensatory mechanisms may help maintain kidney function despite underlying chronic changes. Among the histopathological lesions observed in this study, tubular atrophy (90.86%) and glomerulosclerosis (92.17%) were the predominant findings in biopsies, with a gradual increase in severity with disease progression and CKD stage 1 to 5. Detailed distributions of the semi-quantitative histopathological scores, glomerular summary measures, and selected exclusive lesion patterns are provided in Supplementary Table 1. Previous studies have reported a similar correlation between disease progression and the severity of tubular atrophy and interstitial fibrosis [7, 12, 13].

Interstitial fibrosis and tubular atrophy showed a strong positive correlation (*r* = 0.687; Supplementary Fig. 1), which is consistent with their close structural relationship as components of chronic tubulointerstitial damage. In contrast, tubulitis showed only a modest correlation with tubular atrophy (*r* = 0.302) and no significant correlation with interstitial fibrosis (*r* = 0.094), suggesting that inflammatory activity may coexist with chronic scarring but does not fully parallel it.

46% of the patients that participated in this study had at least one family member who was previously diagnosed with CKDu while 53.2% had no such history, indicating a genetic or common expoures [24]. In addition to these major findings, we also noted that 16.1% of the study participants reported a history of snakebite prior to CKDu diagnosis. However, no significant association between snakebite-associated acute kidney injury and chronic kidney disease was found in prior longitudinal studies [25, 26]. Our findings call for further scrutiny of the diagnostic criteria. Looking at proteinuria in the cohort, 58% of the participants had only 0 to trace levels, which is typical for tubular interstitial nephropathies [15].

A central aim of this study was to determine whether histopathological changes at the time of biopsy were associated with subsequent decline in eGFR. We found that tubulitis was significantly correlated with eGFR decline. However, given the small follow-up cohort and the low correlation coefficients, these findings should be interpreted cautiously as associations rather than robust prognostic indicators. The potential of using morphological changes to predict kidney disease progression has also been investigated in relation to other kidney diseases. For example, individual studies on the predictive potential of the MEST-C score in frequent IgA nephropathy have shown that this is possible over a follow-up period of one year [26]. However, a meta-analysis of the predictive potential of the MEST-C score showed that the development of end-stage kidney disease (ESKD) as a clinical outcome correlated positively with the IFTA in 65% of studies (17/26) [27]. In 30% of studies, however, no association could be found between any MEST-C score parameter and progression to ESKD [27]. Individual kidney biopsies provide a snapshot of changes at the time of biopsy, which is important for diagnosis and classification. Although it would be desirable to confirm the clear prognostic relevance of histological parameters for CKDu treatment, the limited predictive value in our study is not unexpected, as this has not been possible even for common diseases such as IgAN over a longer period of time.

Since only two of the investigated histological lesions provided a prognosis for the progression of the disease, we also took other factors into consideration. In this retrospective study, participants exhibited characteristic exposures to farm work, agrochemical use, well water consumption, and tobacco use, which are not common in other CKD patients. Despite a very similar distribution of possible influencing factors in slowly and rapidly progressing CKDu, the relatively faster progressing group of the study cohort has a slightly higher percentage of agrochemical users ~ 10% when compared to the slowly progressing individuals. The notable shift of the participants from unfiltered well water to tap water and filtered drinking water consumption can be explained by the increased awareness and availability of safe drinking water during the period of 2017 [28]. At the time, the main strategic approaches of the Sri Lankan government to combat CKDu was conduct mass CKDu screening programs and provide safe drinking water to affected communities. A further limitation is that this was a retrospective, biopsy-based cohort with clinicopathological features compatible with CKDu, and therefore some degree of diagnostic heterogeneity and selection bias cannot be excluded. The cohort may not fully represent the broader CKDu population identified through screening programs. In addition, the number of cases with long-term follow-up data was limited, which restricts the strength of inference regarding kidney function decline over time. Electron microscopy was not available, and therefore subtle coexisting glomerular pathology could not be excluded with certainty in all cases. Finally, information on environmental and behavioral exposures was based largely on self-reported data and is therefore subject to recall bias.

Furthermore, as with any biopsy-based study, histopathological assessment captures only a single time point in disease progression. It would be interesting to examine subsequent biopsies to investigate the progression of histopathologic changes.

## Conclusion

In conclusion, our study describes the spectrum of kidney lesions occurring in biopsies from Sri Lanka patients with CKDu. Tubulitis was associated with faster decline in kidney function in the follow-up subcohort. In contrast, pathological changes in the kidneys of patients with CKDu can only be accurately detected by biopsy, while eGFR and creatinine values only reflect the underlying pathological processes to a limited extent. The low eGFR decline observed in the study group may be due to the interventions implemented, such as the provision of safe water, which may mitigate disease progression. 

## Supplementary Information

Below is the link to the electronic supplementary material.

Supplementary Material 1

## Data Availability

Deidentified data used in the current analysis will be made available to researchers after approval of a proposal for their use, according to the rules of the local Ethical Committee. Requests can be sent directly to the corresponding author (nishantha4313@gmail.com).

## Abbreviations

CKDu: Chronic kidney disease of uncertain aetiology
CKD: Chronic kidney disease
ESKD: End-stage kidney disease
eGFR: Estimated glomerular filtration rate
RO: Reverse osmosis
HE: Hematoxylin and eosin
IgG: Immunoglobulin G
IgA: Immunoglobulin A
IgM: Immunoglobulin M
C3: Complement component 3
CKD-EPI: Chronic Kidney Disease Epidemiology Collaboration (equation/formula)
M-ANOVA: Multivariate analysis of variance
SPSS: Statistical Package for the Social Sciences (SPSS Statistics)
IBM: International Business Machines
NY: New York
USA: United States of America
R: R statistical computing environment
v (e.g., v4.2.2): Version
hclust: Hierarchical clustering function in R
ward.D2: Ward’s minimum variance method (Ward.D2 linkage in R)
fviz_nbclust: Cluster number visualization function (factoextra package)
hcut: Hierarchical clustering with cut function (as used in factoextra)
factoextra: R package “factoextra”
pheatmap: R package/function “pheatmap”
rstatix: R package “rstatix”
IF: Interstitial fibrosis
PGF: Periglomerular fibrosis
II: Interstitial inflammation / inflammatory infiltrate
IFTA: Interstitial fibrosis and tubular atrophy
AINu: Acute interstitial nephritis of unknown etiology
IgAN: IgA nephropathy
MEST-C: Oxford MEST-C histologic score: Mesangial hypercellularity (M), Endocapillary hypercellularity (E), Segmental sclerosis (S), Tubular atrophy/interstitial fibrosis (T), Crescents (C)
ESKD: End-stage kidney disease
p: p-value
mg/dL: Milligrams per deciliter
mL/min/year: Milliliters per minute per year
n: Sample size
