# Supplementary material for: A retrospective study of CKDu progression in Sri Lanka: analysis of kidney biopsies and association with risk factors
Source: BMC Nephrol. 2026 Jun 6;27:401. doi: 10.1186/s12882-026-05105-7 (PMC13332587; doi:10.1186/s12882-026-05105-7)
Supplement: Supplementary file 1 — Supplementary Material 1 [file 12882_2026_5105_MOESM1_ESM.docx]

# Supplementary Figures and Tables

**Supplementary Table S1. Detailed histopathologic features of the renal biopsy cohort**

A. Semi-quantitative histopathologic scores

| Histologic parameter | Number of biopsies assessed | Score 0, n (%) | Score 1, n (%) | Score 2, n (%) | Score 3, n (%) |
| --- | --- | --- | --- | --- | --- |
| Tubulitis | 230 | 119 (51.7) | 60 (26.1) | 44 (19.1) | 7 (3.0) |
| Lymphocytic infiltration | 230 | 43 (18.7) | 118 (51.3) | 60 (26.1) | 9 (3.9) |
| Tubular atrophy | 230 | 21 (9.1) | 120 (52.2) | 74 (32.2) | 15 (6.5) |
| Interstitial fibrosis | 230 | 57 (24.8) | 96 (41.7) | 66 (28.7) | 11 (4.8) |
| Global glomerulosclerosis score | 230 | 18 (7.8) | 97 (42.2) | 84 (36.5) | 31 (13.5) |
| Periglomerular fibrosis score | 230 | 134 (58.3) | 94 (40.9) | 2 (0.9) | 0 (0.0) |

B. Glomerular summary

| Parameter | Number of biopsies assessed | Mean ± SD | Range |
| --- | --- | --- | --- |
| Total glomeruli per biopsy | 230 | 14.1 ± 7.1 | 7 to 51 |
| Globally sclerotic glomeruli per biopsy | 230 | 4.9 ± 4.9 | 0 to 38 |
| Globally sclerotic glomeruli (%) | 230 | 34.2 ± 24.6 | 0.0 to 100.0 |

C. Exclusive lesion patterns

| Exclusive histologic pattern | Number of biopsies | Percent of biopsies |
| --- | --- | --- |
| Tubulitis without tubular atrophy and interstitial fibrosis | 4 | 1.7 |
| Tubular atrophy without tubulitis and interstitial fibrosis | 15 | 6.5 |
| Interstitial fibrosis without tubulitis and tubular atrophy | 5 | 2.2 |

Footnote: Histopathologic lesions were scored semi-quantitatively as 0 = absent, 1 = up to 30% of cortical involvement, 2 = 30 to 60%, and 3 = more than 60%. Global glomerulosclerosis percentage was calculated as the proportion of globally sclerotic glomeruli among the total number of glomeruli in each biopsy. Exclusive lesion patterns were defined as presence of the named lesion with absence of the other two listed lesions.


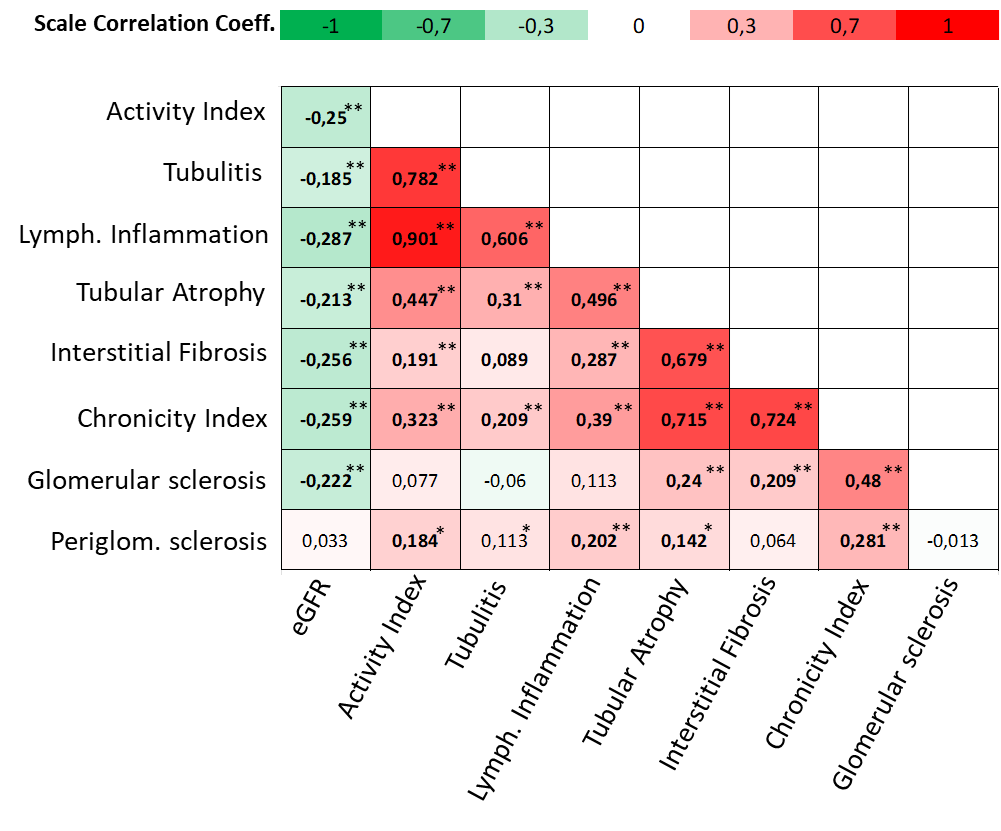


**Supplementary Figure 1:** Heatmap of correlations between severity of histopathologic lesions and eGFR using the total CKD cohort (n=230). Heatmap visualization of pairwise Spearman’s correlation coefficients between the following variables: histological parameters and eGFR. Heatmap colors represent the correlation coefficients (red: positive; green: negative). * p<0.05, ** p<0.01.


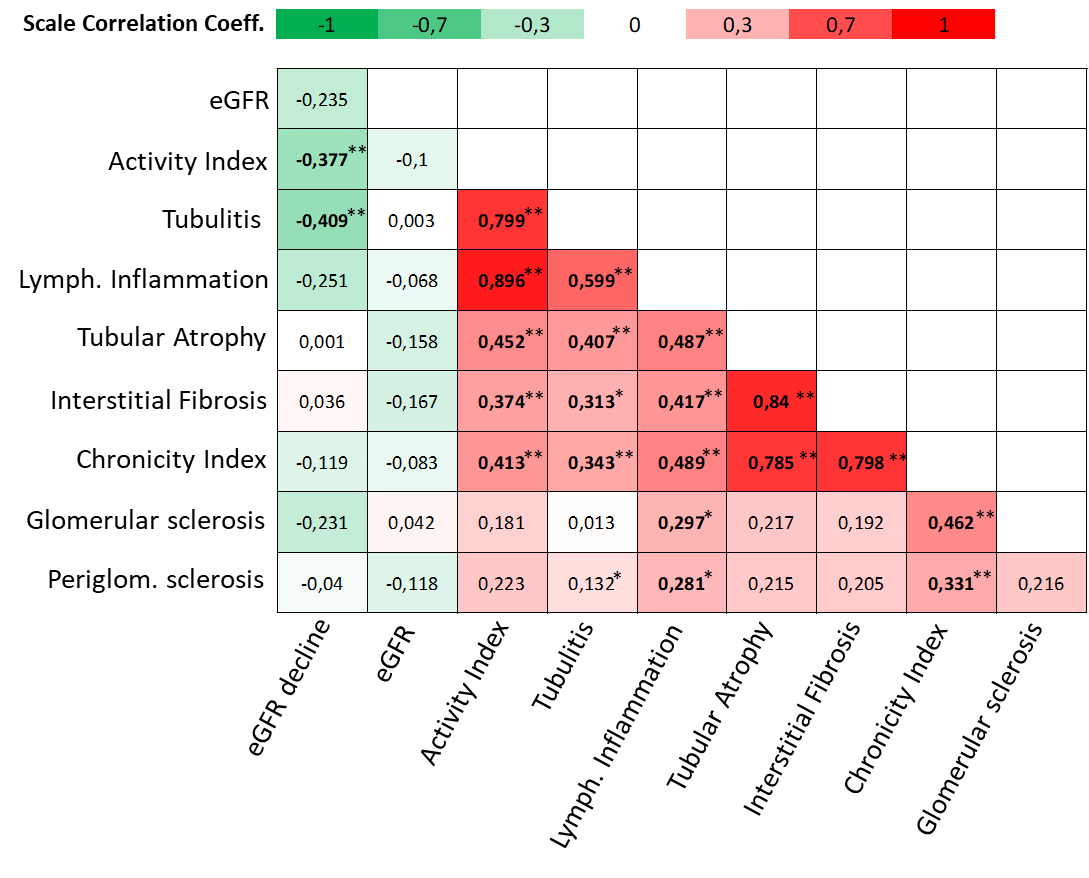


**Supplementary Figure 2:** Heatmap of correlations between severity of histopathologic lesions eGFR and eGFR decline using the follow-up cohort (n=62). Heatmap visualization of pairwise Spearman’s correlation coefficients between the following variables: histological parameters, eGFR, and eGFR decline. Heatmap colors represent the correlation coefficients (red: positive; green: negative). * p<0.05, ** p<0.01.
